# A Simplified Three-Layered Suturing Training Pad for Undergraduate Medical Students: A Technical Note

**DOI:** 10.7759/cureus.47330

**Published:** 2023-10-19

**Authors:** Ioannis Antonopoulos, Andrianos-Serafeim Tzortzis, Evmorfia Pechlivanidou, Theodore Troupis

**Affiliations:** 1 Department of Anatomy, Medical School, National and Kapodistrian University of Athens, Athens, GRC; 2 Emergency Department, East and North Hertfordshire NHS Trust, Lister Hospital, Stevenage, GBR; 3 Department of Hygiene, Epidemiology and Medical Statistics, Medical School, National and Kapodistrian University of Athens, Athens, GRC

**Keywords:** basic surgical skills, medical training, surgical education, suturing training, suturing model

## Abstract

Surgical training is a long process that requires a lot of commitment and effort. Basic surgical techniques are the foundation of every procedure, with suturing being one of them. Hence, it is of great importance for aspiring young surgeons to practice and develop their suturing skills. Quite many kinds of suturing training models have been used and proposed worldwide, ranging from commercial silicone pads to meat leftovers and various fruits. We have developed our own, simplified, and low-cost suturing training pad that consists of three layers and is based on the combined use of silicone sponge sheet and polyurethane foam. It is quite durable and elastic and has been applied in three suturing training workshops so far. For this reason, we would like to present our experience of a low-cost but effective way of promoting and achieving further surgical excellence.

## Introduction

Sutures play a crucial role in facilitating optimum wound healing subsequent to a range of surgical interventions. Improperly positioned sutures not only contribute to delayed wound healing but can also result in tissue damage. Thus, it is imperative for young surgeons to possess proficient suturing skills [1]. It is also quite important that medical students should have both the theoretical background and the practical experience before suturing a wound on a patient.

Worldwide, a wide variety of suturing training models have been used and proposed, including commercial synthetic pads, meat leftovers (usually pig or chicken skin), and various fruits, usually bananas and oranges [2]. Kumaresan and Karthikeyan proposed the use of a simple model made from an orange peel, putty impression material, and Plaster of Paris for use by dental students [3].

In many medical schools and training centers, the acquisition of commercially accessible suturing training pads may be impeded due to financial constraints. The utilization of pig or chicken skin and leftover meat might be a valuable alternative. However, the acquisition and preservation of these resources may provide certain challenges, especially in tropical regions [4], and their use is something that has to be both well controlled and acceptable from the competent authorities of each region. Finally, these meat leftovers are not reusable by the trainee.

A recent study by Gonzalez-Navarro et al. showed that except for the pigskin, sponges are effective and well-received training pads by undergraduate medical students too [5]. According to the findings of this study, the combination of resistance, elasticity, and uniformity is required for an effective suturing training model.

Taking all these into consideration, we designed a simplified and easy-to-assembly suturing training pad for undergraduate medical students, which would be both cost effective and reusable.

## Technical report

**Figure 1:**
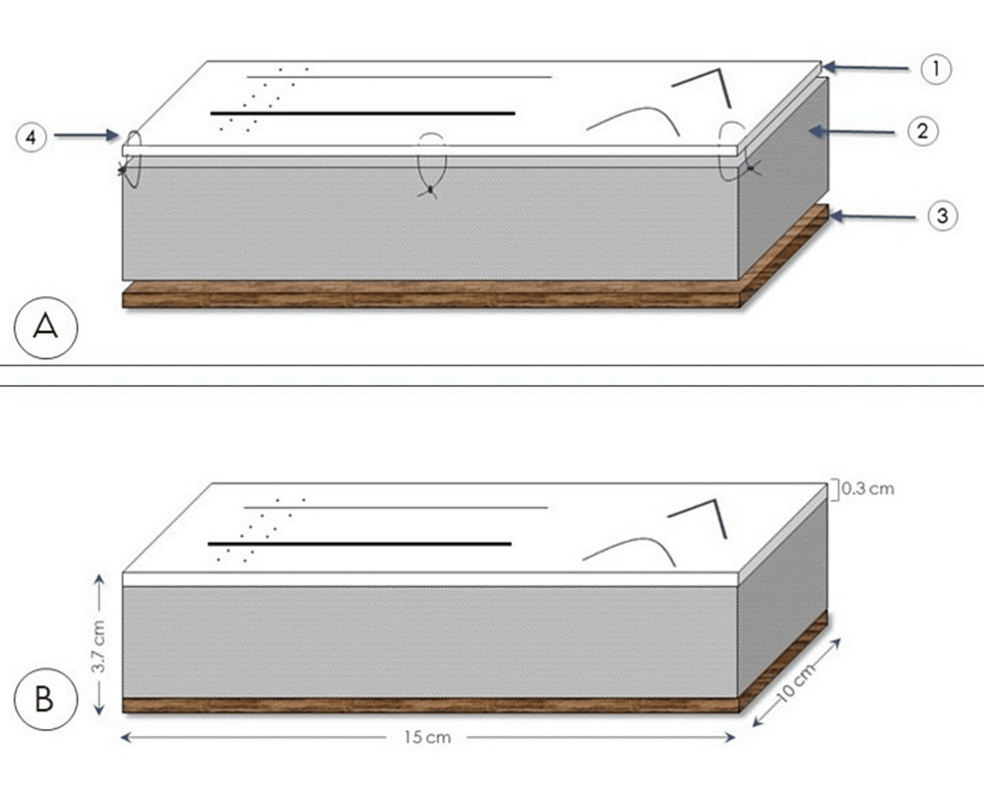
Illustration of the proposed training pad. A. Depiction of the three layers: 1) the silicone foam sheet with the various incisions representing the skin (L1), 2) the sponge representing the subcutaneous tissue (L2), 3) the last wooden layer (L3), 4) the sutures used to reinforce the connection between the first and second layers. B. The dimensions of the training pad.

**Figure 2:**
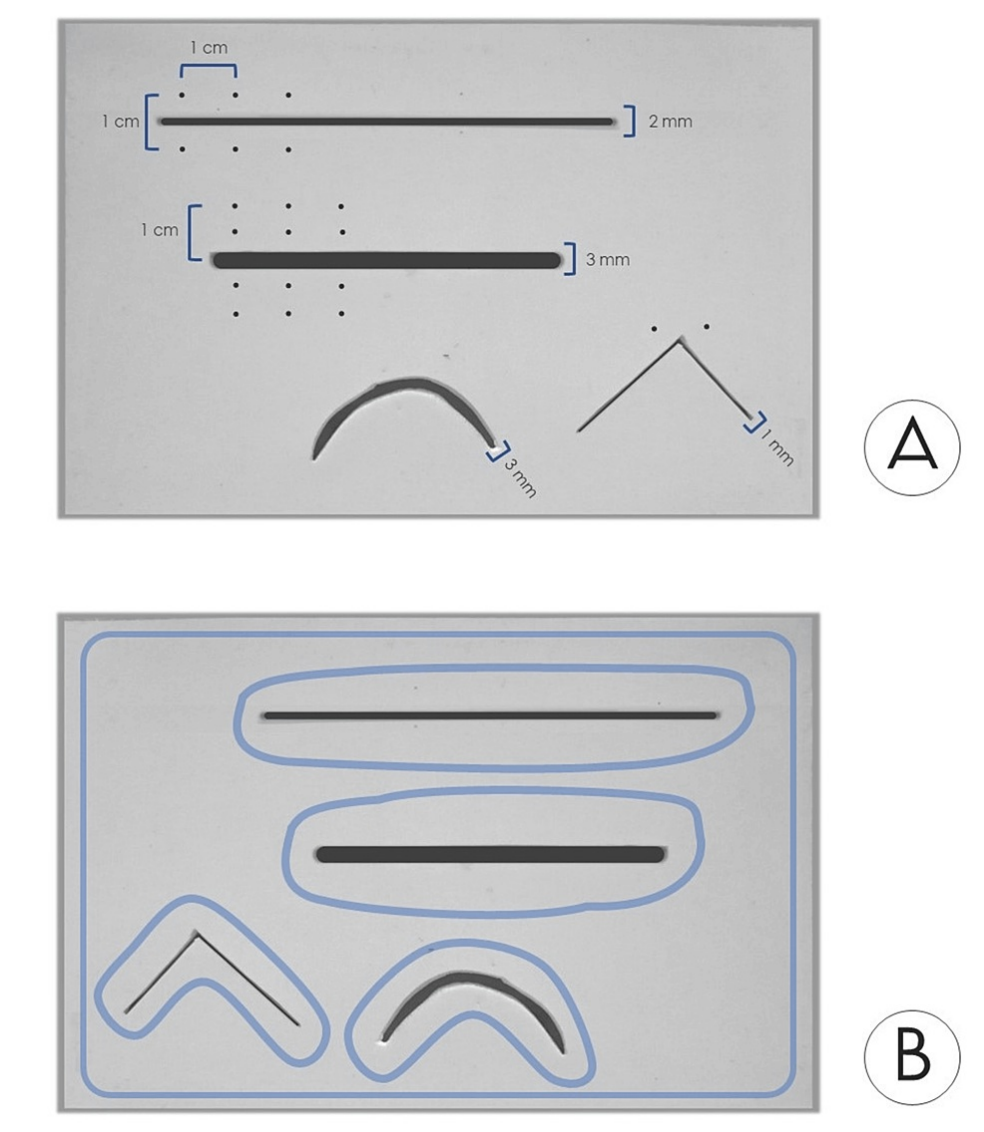
Preparation of the silicone foam sheet (L1). A. The superior surface of L1 with the four incisions. B. The L1 inferior surface; the sites of liquid glue application have been marked with blue.

Our training pad consists of three different layers: the superficial layer (L1) that represents the skin, an intermediate layer (L2) that represents the subcutaneous soft tissue, and a last layer (L3) that is actually the base of the model and provides stability to it (Figure 1A). For the L1, we chose to use a silicone sponge (foam) sheet of 3 mm thickness. The reason for this choice is both the flexibility and elasticity of this material, as well as its soft texture. It is also a relatively easy-to-find material that is durable in tension and can be used multiple times by the trainee. The thickness of the silicon foam sheet used initially for the L1 was approximately 2 mm, which was similar to the mean (thin) skin thickness of an adult human [6]. However, after some trials with this size, we preferred to use a sheet of 3 mm thickness to achieve a greater durability of L1. On this sheet, we made four incisions: a narrow (2 mm wide) straight one for simple sutures, a wide (3 mm wide) straight for mattress suture, a curved one (2 mm wide), and a 90° corner incision (1 mm wide) for training in suturing corners (Figure 2A). The silicone sheet used was white in color, although the sheets are available in other colors as well (representing the distribution of skin tone diversity in the training location) that could be used to achieve a more realistic result.

The L2 in our model is a polyurethane foam sponge. It can be of various thicknesses; however, we propose the use of a 3-cm-thick sponge (53 kg/m^3^ density). The connection of L1 with L2 was achieved by applying an ethyl cyanoacrylate based liquid glue (we have used the one produced by UHU GmbH & Co.KG, Bühl, Germany) on the undersurface of the L1 in the sites shown in Figure 2B, and then constant force (about 20 Newtons) uniformly distributed over the surface was applied for 10 minutes. For further reinforcement of the junction, we applied 3-0 nylon sutures in the four corners of the pad (Figure 1A; 4), but this step is optional. As for the L3, we used a wooden piece of 4 mm thickness to increase the overall weight and stability of the training pad. To achieve a greater degree of stability, we applied a strip of polyethylene-based anti-slip tape to the inferior surface of the L3. The dimensions of our training pad after its assembly are shown in Figure 1B.

Both the silicon foam sheet and the polyurethane foam are easy to find and can be bought in pieces of square meters and then cut into 10x15cm for the construction of the suturing pad in big quantities. The whole assembly process duration ranged from 25 to 35 minutes. The cost of the production of this model in Athens, Greece, in 2021 was calculated to be up to 8 USD (using the currency exchange rate for September 2023 on google.com/finance).

**Figure 3:**
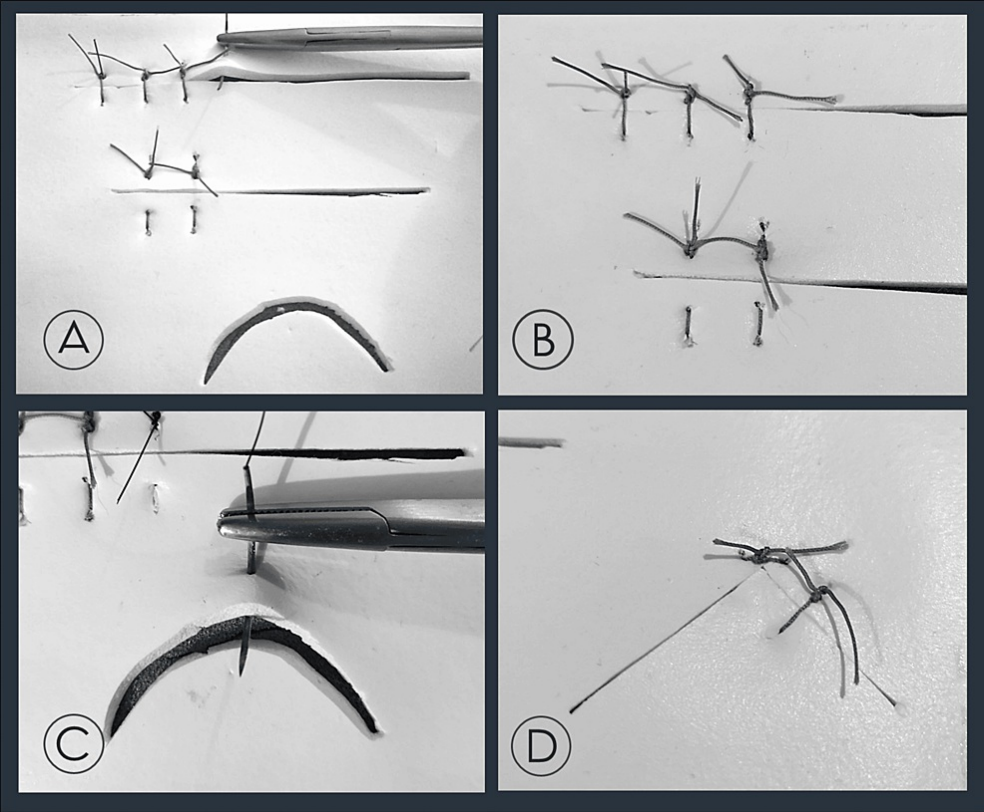
The suturing training pad in use. A. Practicing the simple interrupted sutures; B. Simple sutures on the narrow incision and vertical mattress sutures in the wider incision below; C. Practicing the simple sutures on the semi-lunar incision; D. Corner sutures.

Any type of sutures can be used; however, we do strongly recommend the use of medium size sutures (2-0, 3-0) with a round or tapered needle to achieve both better handling and less “damage” of the L1, leading to greater durability of the whole training pad. The final result as well as the behavior of the silicone foam (L1) and the suturing training pad as an entity is shown in Figure 3.

## Discussion

In the existing literature, many alternatives for skin simulation in terms of suturing skills training have been described. As previously stated, the range of training models available is extensive, including synthetic skin, diverse fruits, pork skin, and even pieces of fabric. To the best of our knowledge, there is no previous description of the combinational use of silicone sponge and polyurethane foam for the construction of a simulation pad for suturing training.

Silva et al. have described an "alternative model of silicone" for surgical teaching purposes. However, the researchers opted for a combination of cornstarch and silicone-based sealant instead of utilizing silicone sponge sheets and polyurethane foam [7]. Furthermore, the researchers implemented a sequential approach by constructing each of the three layers separately and subsequently combining them into a training pad. In contrast, our approach involved the purchase of the three distinct layers followed by a simple assembly procedure to create our suturing model.

To address the educational requirements of our medical students, we have manufactured a total of 80 suturing training pads. It is noteworthy to emphasize that these tools have been effectively employed in three suturing training workshops targeting undergraduate students within a one-year timeframe, resulting in their reuse on eight separate occasions. Furthermore, the vast majority of trainees have shown a positive reception toward these tools. More specifically, a total of 410 (50.59% females; mean age: 21 years; SD: 1.43) undergraduate medical students participated in those workshops. A 5-scale Likert anonymous questionnaire assessed workshop participants' opinions. Regarding the question rating the training pad described, 301 (73.41%) expressed that the pad was exceptional, as shown by a perfect rating of 5 out of 5 on the Likert scale, while 88 (21.46%) rated the pad as very good (4/5).

## Conclusions

This technical report provides a detailed description of an affordable and easy-to-assemble suturing training pad. We believe that the use of such materials has the potential to improve the suturing skills of medical students, whether they are produced and employed on a broad scale by educational institutions or utilized individually by medical students.
